# S100 calcium-binding protein A9 promotes skin regeneration through toll-like receptor 4 during tissue expansion

**DOI:** 10.1093/burnst/tkad030

**Published:** 2023-10-31

**Authors:** Yu Zhang, Yajuan Song, Jing Du, Wei Liu, Chen Dong, Zhaosong Huang, Zhe Zhang, Liu Yang, Tong Wang, Shaoheng Xiong, Liwei Dong, Yaotao Guo, Juanli Dang, Qiang He, Zhou Yu, Xianjie Ma

**Affiliations:** Department of Plastic Surgery, Xijing Hospital, Fourth Military Medical University, No.127 Changle West Road, Xi’an, Shaanxi Province 710032, China; Department of Plastic Surgery, Xijing Hospital, Fourth Military Medical University, No.127 Changle West Road, Xi’an, Shaanxi Province 710032, China; Department of Plastic Surgery, Xijing Hospital, Fourth Military Medical University, No.127 Changle West Road, Xi’an, Shaanxi Province 710032, China; Department of Plastic Surgery, Xijing Hospital, Fourth Military Medical University, No.127 Changle West Road, Xi’an, Shaanxi Province 710032, China; Department of Plastic Surgery, Xijing Hospital, Fourth Military Medical University, No.127 Changle West Road, Xi’an, Shaanxi Province 710032, China; Department of Plastic Surgery, Xijing Hospital, Fourth Military Medical University, No.127 Changle West Road, Xi’an, Shaanxi Province 710032, China; Department of Plastic Surgery, Xijing Hospital, Fourth Military Medical University, No.127 Changle West Road, Xi’an, Shaanxi Province 710032, China; Department of Plastic Surgery, Xijing Hospital, Fourth Military Medical University, No.127 Changle West Road, Xi’an, Shaanxi Province 710032, China; Department of Plastic Surgery, Xijing Hospital, Fourth Military Medical University, No.127 Changle West Road, Xi’an, Shaanxi Province 710032, China; Department of Plastic Surgery, Xijing Hospital, Fourth Military Medical University, No.127 Changle West Road, Xi’an, Shaanxi Province 710032, China; Department of Plastic Surgery, Xijing Hospital, Fourth Military Medical University, No.127 Changle West Road, Xi’an, Shaanxi Province 710032, China; Department of Plastic Surgery, Xijing Hospital, Fourth Military Medical University, No.127 Changle West Road, Xi’an, Shaanxi Province 710032, China; Department of Plastic Surgery, Xijing Hospital, Fourth Military Medical University, No.127 Changle West Road, Xi’an, Shaanxi Province 710032, China; Department of Plastic Surgery, Xijing Hospital, Fourth Military Medical University, No.127 Changle West Road, Xi’an, Shaanxi Province 710032, China; Department of Plastic Surgery, Xijing Hospital, Fourth Military Medical University, No.127 Changle West Road, Xi’an, Shaanxi Province 710032, China; Department of Plastic Surgery, Xijing Hospital, Fourth Military Medical University, No.127 Changle West Road, Xi’an, Shaanxi Province 710032, China

**Keywords:** S100 calcium-binding protein A9, Skin, Soft tissue expansion, Mechanical stretch, Regeneration

## Abstract

**Background:**

In plastic surgery, tissue expansion is widely used for repairing skin defects. However, low expansion efficiency and skin rupture caused by thin, expanded skin remain significant challenges in promoting skin regeneration during expansion. S100 calcium-binding protein A9 (S100A9) is essential in promoting wound healing; however, its effects on skin regeneration during tissue expansion remain unclear. The aim of the present study was to explore the role of S100A9 in skin regeneration, particularly collagen production to investigate its importance in skin regeneration during tissue expansion.

**Methods:**

The expression and distribution of S100A9 and its receptors—toll-like receptor 4 (TLR-4) and receptor for advanced glycation end products were studied in expanded skin. These characteristics were investigated in skin samples of rats and patients. Moreover, the expression of S100A9 was investigated in stretched keratinocytes *in vitro*. The effects of S100A9 on the proliferation and migration of skin fibroblasts were also observed. TAK-242 was used to inhibit the binding of S100A9 to TLR-4; the levels of collagen I (COL I), transforming growth factor beta (TGF-β), TLR-4 and phospho-extracellular signal-related kinase 1/2 (p-ERK1/2) in fibroblasts were determined. Furthermore, fibroblasts were co-cultured with stretched S100A9-knockout keratinocytes by siRNA transfection and the levels of COL I, TGF-β, TLR-4 and p-ERK1/2 in fibroblasts were investigated. Additionally, the area of expanded skin, thickness of the dermis, and synthesis of COL I, TGF-β, TLR-4 and p-ERK1/2 were analysed to determine the effects of S100A9 on expanded skin.

**Results:**

Increased expression of S100A9 and TLR-4 was associated with decreased extracellular matrix (ECM) in the expanded dermis. Furthermore, S100A9 facilitated the proliferation and migration of human skin fibroblasts as well as the expression of COL I and TGF-β in fibroblasts via the TLR-4/ERK1/2 pathway. We found that mechanical stretch-induced S100A9 expression and secretion of keratinocytes stimulated COL I, TGF-β, TLR-4 and p-ERK1/2 expression in skin fibroblasts. Recombined S100A9 protein aided expanded skin regeneration and rescued dermal thinning in rats *in vivo* as well as increasing ECM deposition during expansion.

**Conclusions:**

These findings demonstrate that mechanical stretch promoted expanded skin regeneration by upregulating S100A9 expression. Our study laid the foundation for clinically improving tissue expansion using S100A9.

## Graphical Abstract

**Graphical Abstract:**
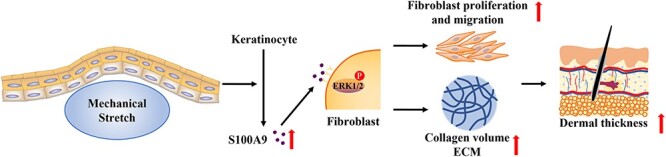

Highlights

- Dermal thinning could cause skin rupture which inhibited skin regeneration during tissue expansion.
- S100A9, which was up-regulated and secreted in the expanded epidermis, elevated collagen synthesis and enhanced ECM deposition in expanded skin via TLR-4 and activation of ERK1/2 protein, causing an increase in dermal thickness.
- We showed that S100A9 might be a possible therapeutic target for dermal thinning during skin expansion.

## Background

Skin tissue expansion has been widely used for repairing skin defects and congenital malformations [1]. Neumann first reported this technique in 1957 for repairing ear deformities [2]. During tissue expansion, the mechanical stretch to the normal skin caused by the continuous inflation of the expander generates new skin that resembles the adjacent tissue in colour, texture and lustre [3]. Proliferation and growth of skin cells, elastic stretching of the skin and translocation of surrounding skin all contribute to the generation of new skin. Among these, cell proliferation and growth are the most important factors for skin regeneration. However, the two major challenges for promoting skin regeneration during tissue expansion are low expansion efficiency and skin rupture caused by thin expanded dermis [4–6]. Elucidating the mechanism of skin regeneration induced by mechanical stretch may resolve the aforementioned issues.

Tissue expansion frequently results in dermal thinning, which may further lead to skin rupture [7, 8]. Therefore, it is essential to identify the factors contributing to collagen production; this may aid in promoting dermal thickening and benefit the clinical application of this technique. Recent studies have shown that the S100 calcium-binding protein A9 (S100A9) protein and other S100 protein family members play crucial roles in wound repair and extracellular matrix (ECM) production [9–11]. S100A9, also known as calprotectin [12], is involved in wound healing and fibrosis of the kidney [13–15], lung [16] and skin [17, 18] by binding to receptors such as toll-like receptor 4 (TLR-4) [15] and receptor for advanced glycation end products (RAGE) [14]. Our previous study revealed increased mRNA expression of S100A9 in expanded rat skin [19]. Further, studies using the single-cell RNA sequencing (RNA-seq) clustering analysis have shown elevated S100A9 expression in mouse epidermis following skin expansion [20, 21]. However, the effects of S100A9 on expanded skin regeneration during tissue expansion remain unknown.

In the present study, to investigate the role of S100A9 in skin regeneration during tissue expansion, we examined S100A9 distribution in expanded human and rat skin tissues. We further explored its effects on skin regeneration, particularly collagen production, which is a novel candidate option for accelerating skin regeneration during tissue expansion in plastic surgery.

## Methods

### Rat scalp expansion model establishment

Adult male Sprague–Dawley rats weighing 200–250 g were purchased from the Fourth Military Medical University’s Animal Center. Rats were housed in a clean, temperature-controlled environment with a 12-h light/dark cycle, fed a standard laboratory diet and provided *ad libitum* access to water. All animal experiments were performed according to the Guidelines for the Care and Use of Laboratory Animals by the National Research Council (US) Committee [8th edition; Washington (DC): National Academies Press (US); 2011]. All experimental procedures were approved by the ethics committee of the Fourth Military Medical University (IACUC-20191207); every effort was made to reduce the number and suffering of animals. All animal experiments were conducted in the Laboratory of the Department of Plastic Surgery at Xijing Hospital, Fourth Military Medical University.

The rats were randomly divided into two groups: control (n = 8) and expanded (n = 8). The rat scalp expansion model was created using our previously described procedures [22]. Briefly, all rats were anaesthetised with 4% isoflurane (Keyuan Pharma, Shandong, China) in oxygen at 2 l/min via a face mask to minimize suffering. The rat scalp expansion model was constructed using silicone expanders (1 ml, Wanhe, Guangzhou, China). The 1.0 × 1.0 cm^2^ square area in the middle of the expanded flap was tattooed for tracing the expanding areas. The expander was implanted beneath the scalp, following which 1 ml of saline was injected into it. The control group was a sham expansion group implanted with a silicone sheet. Expanders in the expanded group were routinely inflated (1 ml each time, twice a week), whereas the control group received no injections. The first saline injection was designated as day 0. Tissues were harvested on day 28 and used for mRNA and protein analysis. Animals that completed the study were intraperitoneally euthanised using large amounts of pentobarbital.

Human tissue samples including 11 expanded skin and 4 normal skin biopsies were collected from various volunteers. Expanded skin samples had been expanded for ~3 months before collection. The control skin comprised normal skin samples. Table S1 mentions the clinical data and sample information (including sex, age, expander placement area, injected saline volumes and sampling time) of patients. All patients provided their written informed consent for using skin samples, and the study was authorized by the ethics committee of Xijing Hospital, Fourth Military Medical University (KY20192155-C-1).

### Histological analysis

The harvested skin samples were fixed in 4% paraformaldehyde before being embedded in paraffin. Sections of 4-μm thick were stained with haematoxylin and eosin (H&E). Masson’s trichrome and Sirius red staining were used to assess the dermal thickness, collagen content and collagen types.

### RNA-seq

Our previous study described the procedures for RNA-seq of expanded rat skin [19]. Briefly, the harvested tissues were subjected to total RNA extraction, quality control and library preparation procedures. Paired-end RNA-seq analysis was performed by Annoroad Gene Technology Co. Ltd (http://www.annoroad.com/) using Illumina HiSeq 4000 [23]. The sequencing reads were aligned to a reference sequence using Bowtie2 (v2.2.5) (Maryland, USA); the gene expression levels were calculated using the RSEM software package (v1.2.12). The downstream analysis excluded mRNAs that were not detected in any sample (read count <1). The differentially expressed genes were discovered using the R language.

### Immunofluorescence staining

After being deparaffinized and rehydrated in varying alcohol concentrations, the tissue sections were placed in a sodium citrate solution at 96°C for 20 min before being blocked for 1.5 h in phosphate-buffered solution (PBS) containing 5% bovine serum albumin and incubated with rabbit anti-rat S100A9 (1 : 50, Proteintech, Wuhan, China), rabbit anti-rat TLR-4 (1 : 50, Abcam, Cambridge, UK), rabbit anti-rat RAGE (1 : 50, Abcam, Cambridge, UK) and rabbit anti-human S100A9 (1 : 50, Proteintech, Wuhan, China) overnight at 4°C in humid chambers. The sections were incubated in the same solution for 2 h at room temperature (22–24°C) on the following day before incubation with a goat anti-rabbit Alexa Fluor 488 secondary antibody (1 : 200, Invitrogen, Carlsbad, CA, U.S.A.) or goat anti-rabbit Alexa Fluor 594 secondary antibody (1 : 200, Invitrogen, Carlsbad, CA, U.S.A.) at room temperature for 1 h. The nuclei were stained with 4′,6-diamidino-2-phenylindole at 1 : 1000 dilution (Invitrogen, Carlsbad, CA, USA).

### Cell culture

HaCaT cells and human skin fibroblasts were cultured in Dulbecco’s modified Eagle’s medium (Gibco, Grand Island, NY, USA). All culture media were supplemented with 10% fetal bovine serum (BI, Israel), 100 U/ml penicillin and 100 μg/ml streptomycin and cultured in a 5% CO_2_ humidified atmosphere at 37°C.

### Application of cyclic stretch

HaCaT cells were seeded on a silicone chamber coated with fibronectin (100 μg/ml) at a density of 10^5^ cells/cm^2^. A uniaxial sinusoidal stretch of 12% at a frequency of 15 cycles/min with sine wave was applied using a stretching apparatus (model ST-140, STREX Inc. Osaka, Japan) driven by a computer-controlled stepping motor, as previously demonstrated [24]. The relative elongation of the silicone membrane was uniform across the entire membrane area. Control cells were cultured in the same chambers under static conditions. The cells and their proteins were collected at 0, 3, 6, 12 and 24 h after stretching.

### Enzyme-linked immunosorbent assay

After stretching, the S100A9 concentrations in the supernatants of cultured HaCaT cells were collected at 0, 3, 6, 12 and 24 h and measured using an S100A9 enzyme-linked immunosorbent assay (ELISA) kit (JL19159, J&L, Shanghai, China) according to the manufacturer’s instructions.

### Cell counting kit-8 assay of fibroblasts

The effects of S100A9 on human skin fibroblast proliferation were evaluated using the cell counting kit-8 (CCK-8) assay (Abmole Laboratories, Mashiki, Japan). Briefly, fibroblasts were seeded in 96-well plates and starved for 12 h. Then, the medium was supplemented with culture medium with or without S100A9 (0, 200, 500 and 1000 ng/ml); the cells were tested with CCK-8 reagent at 0, 24, 48 and 72 h.

### 5-Ethynyl-2′-deoxyuridine assay of fibroblasts

The 5-ethynyl-2′-deoxyuridine (EdU) assay was used to assess fibroblast proliferation. Human skin fibroblasts were cultured in the 24-well plates at a density of 1 × 10^5^ cells/well. After 24 h, S100A9 solutions of varying concentrations (0, 200, 500 and 1000 ng/ml) were added. Each group had three parallel wells. At 0 and 24 h, the cells were cultured with 200 μL of 1× EdU medium (Beyotine, China) for 4 h and fixed with 200 μL of cell-fixation solution (PBS containing 4% polyformaldehyde) for 15 min at room temperature. Subsequently, the cells were washed and rinsed with 200 μL of PBS containing 0.5% TritonX-100 (Beyotine, China) for 10 min before staining with 1× Apollo staining reaction solution (Beyotine, China) for 30 min in the dark. The cells were treated with 100 μL of 1× Hoechst 33342 reaction solution (Beyotine, China) for 30 min before being sealed with 200 μL of the anti-fluorescence quenching agent. Approximately 6–10 fields of view were randomly selected for each well and photographed using a fluorescence microscope (Nikon ECLIPSE Ts2R, Nikon, Japan).

### Migration assay of fibroblasts

Human skin fibroblasts were seeded in six-well plates. After cell density reached 90% confluency, the cells were starved for 12 h. The cell monolayer was scratched and lined with a pipette tip before being treated with S100A9 (500 ng/ml) cultured in 2% Dulbecco’s modified Eagle’s medium for 24 h. The cells were washed with PBS and cultured in serum-free medium at 0 and 24 h. The distance between the cells on both sides of the scratch was calculated using ImageJ software (1.6.0 20). The tests were performed at least three times.

Moreover, the transwell assay was used to evaluate the migration ability of fibroblasts. The transwell cell culture chamber contains a polycarbonate membrane with a pore size of 4 μm (Corning, NY, U.S.A.). The cells (2 × 10^5^ cells/well) were seeded into the upper chamber in 200 μl serum-free medium; 500 μl complete medium containing 10% fetal bovine serum was placed in the lower chamber as a chemoattractant. Following incubation for 24 h, cells were treated with or without S100A9 (500 ng/ml) for 0 and 24 h. The membranes were fixed in 4% paraformaldehyde for 15 min and stained with crystal violet for 20 min. The cells on the upper side of the membranes were gently wiped away with a cotton swab; cells on the lower side of the membranes were observed and photographed using a microscope (Nikon ECLIPSE Ts2R, Nikon, Japan).

### Quantitative polymerase chain reaction

Total RNA was extracted from the rat scalp samples and cells using TRIzol (Invitrogen, Carlsbad, CA, USA) and reverse transcribed into cDNA with the PrimeScript™ RT reagent kit (No. RR047A, TaKaRa, Dalian, China) and TB Green™ Premix Ex Taq™ (No. RR037A, TaKaRa, Dalian, China). A quantitative polymerase chain reaction (qPCR) analysis was performed using Bio-Rad CFX Manager 3.0. The mRNA expression levels were measured using the 2^−ΔΔCt^ (threshold cycle) method [25]. The primer sequences are listed in Table S2 (see online supplementary material). The following genes were included: rat S100A9, rat collagen I (COL I), rat transforming growth factor beta (TGF-β), human S100A9, human COL I and human TGF-β. Glyceraldehyde-3-phosphate dehydrogenase (GAPDH) was used as an internal reference when evaluating gene expression.

### Western blot

The proteins of tissue samples and cells were lysed in radioimmunoprecipitation assay buffer (NCM Biotech, Suzhou, China). A bicinchoninic acid assay (BCA) kit (CWBIO Biotech, Beijing, China) was used to quantify protein concentrations. Proteins were separated using sodium dodecyl sulphate–polyacrylamide gel electrophoresis (NCM Biotech, Suzhou, China) and transferred to polyvinylidene difluoride membranes (Millipore, Billerica, USA). After blocking for 1 h in PBS containing 5% bovine serum albumin, the membranes were incubated with the following primary antibodies at 4°C overnight: rabbit anti-rat S100A9 (1 : 1000, Proteintech, Wuhan, China), rabbit anti-rat TLR-4 (1 : 200, Abcam, Cambridge, UK), rabbit anti-rat RAGE (1 : 1000, Abcam, Cambridge, UK), rabbit anti-rat COL I (1 : 2000, Proteintech), rabbit anti-rat TGF-β (1 : 1000, Proteintech, Wuhan, China), rabbit anti-rat extracellular signal-related kinase 1/2 (ERK1/2) (1:200, Santa Cruz, Texas, U.S.A.), rabbit anti-rat phospho-ERK 1/2 (p-ERK1/2) (1 : 200, Santa Cruz, Texas, USA) and rabbit anti-rat GAPDH (1:5000, Proteintech, Wuhan, China). After washing three times in Tris-buffered saline with 0.1% Tween-20, the membranes were incubated with either goat anti-rabbit secondary antibody (CW0103, CWBIO, Jiangsu, China) or goat anti-mouse secondary antibody (CW0102S, CWBIO, Jiangsu, China) for 1 h. The membrane was then washed three times before visualization using a biological image shooting software (Tanon 4600, Tanon, Shanghai, China).

### Intervention of S100A9 and TAK-242 on fibroblasts

The human skin fibroblasts were seeded in 6-well plates. After the cell density reached 90% confluency, the cells were treated with PBS, S100A9 (500 ng/ml), TAK-242 (1 μM, Abmole, Houston, TX, USA) or S100A9 + TAK-242 (500 ng/ml S100A9 + 1 μM TAK-242). After 24 h, the cells were collected and prepared for qPCR and western blot assay.

### S100A9 small interfering RNA transfection

The HaCaT cells were plated at 40–50% density and grown to ~75% confluency. On the following day, the cells were treated with 20 nM negative control small interfering RNA (siRNA) (FAM-siRNA) or S100A9 siRNAs dissolved in a non-serum medium according to the manufacturer’s protocol (Sangon Biotech, Shanghai, China). The S100A9 siRNA sequences were as follows, sense strand 5’-CAUCAACACCUUCCACCAAUATT-3′ and anti-sense strand 5’-UAUUGGUGGAAGGUGUUGAUGTT-3′. The cells and their proteins were collected 24 h after transfection. Subsequently, qPCR and western blot were applied to investigate the effects of S100A9 siRNA.

### Co-culture of human fibroblasts and supernatants of HaCaT cells

The human skin fibroblasts were co-cultured with normal HaCaT cell culture supernatants, stretched HaCaT cell culture supernatants, negative control siRNA + stretched HaCaT cells, and S100A9 siRNA + stretched HaCaT cells, respectively. These fibroblasts’ proteins were collected 24 h after co-culture and used to test the expression of COL I, TGF-β, TLR-4, ERK1/2, and p-ERK1/2.

### Subcutaneous injection of S100A9 protein and TAK-242 (TLR-4 inhibitor) into expanded rat skin

For animal experiments, rats were randomly divided into four groups (n = 6): the PBS-injected expanded group (injected with 100 μl of PBS into expanded skin); S100A9-injected expanded group (injected with 100 μl of 5 μg/ml recombinant S100A9 protein into expanded skin according to a previous study [26]); TAK-242–injected expanded group (injected with 0.5 μg of TAK-242 into expanded skin); and S100A9 + TAK-242–treated group (injected with 100 μl of 5 μg/ml recombinant S100A9 protein into expanded skin 6 h after 0.5 μg of TAK-242 injection). Each injection was administered into the expanded flaps. PBS injection into the skin was considered a control. TLR-4 was inhibited using TAK-242 (S100A9 receptor). PBS, S100A9, and TAK-242 were subcutaneously injected into expanded skin at the same time as saline injections into expanders (twice per week, 1 ml each time). The operations and biopsy times were the same as previously mentioned.

### Statistical analyses

All experiments were performed at least three times. All data are presented as the mean ± SD values. Statistical analyses of differences between groups were performed using a Student’s unpaired t-test, one-way analysis of variance (ANOVA), or two-way ANOVA (GraphPad Prism 6.0). Following one-way or two-way ANOVA, we performed the *post hoc* test (Tukey’s test). A *p* value of < 0.05 (^*^), < 0.01 (^*^^*^), or < 0.001 (^*^^*^^*^) was used to determine statistical significance.

## Results

### Increased expression of S100A9 and its receptor TLR-4 was accompanied by decreased ECM in the expanded dermis

**Figure 1:**
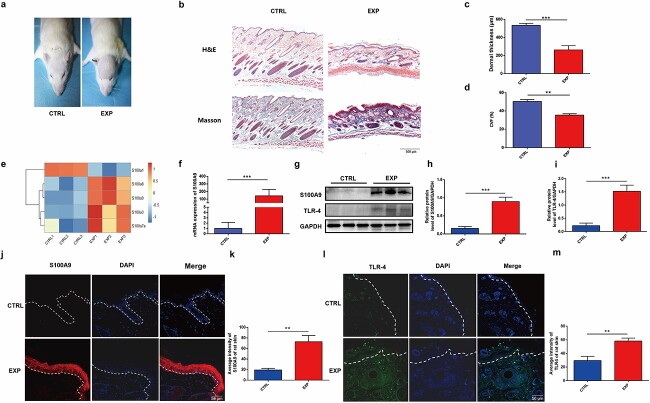
Increased expression of S100A9 and its receptor toll-like receptor 4 (TLR-4) was accompanied by decreased extracellular matrix (ECM) deposition in the expanded rat skin. (**a**) Images of rats from the control group (CTRL) and expanded group (EXP). (**b**) Haematoxylin and eosin (H&E) staining showed thinner dermis in the expanded skin (×40 magnification), and Masson’s trichrome staining showed less collagen content in the expanded skin (×40 magnification). (**c**) Quantification of dermal thickness between expanded and control skin (n = 4). (**d**) Quantification of collagen content in expanded and control skin (n = 3). (**e**) The heat map demonstrated increased mRNA expression of S100A9 in the expanded rat skin (n = 3). (**f**) qPCR analysis confirmed increased mRNA expression of S100A9 in expanded rat skin (n = 7). (**g**) Western blot analysis showed the protein expression of S100A9 and TLR-4 in expanded rat skin. (**h**) Quantification of relative protein levels of S100A9 between the expanded and control rat skin (n = 3). (**i**) Quantification of relative protein levels of TLR-4 between the expanded and control rat skin (n = 3). (**j**) Immunofluorescence labelling of S100A9 in expanded rat epidermis. (**k**) Quantification of the average intensity of S100A9 in expanded and control rat epidermis (n = 8). (**l**) Immunofluorescence labelling of TLR-4 in expanded rat epidermis. (**m**) Quantification of the average intensity of TLR-4 in expanded and control rat epidermis (n = 8). ^*^^*^p < 0.01, ^*^^*^^*^p < 0.001. *S100A9* S100 calcium-binding protein A9, *qPCR* quantitative polymerase chain reaction, *CVF* collagen volume fraction, *GAPDH* Glyceraldehyde-3-phosphate dehydrogenase

Mechanical stretch caused significant alterations in dermal thickness and collagen content. The macroscopic appearance of rats from the control and expanded groups are shown in Figure 1a. H&E staining images showed that the dermis was thinner in the expanded skin than in the control skin (244.00 ± 23.30 μm *vs* 533.90 ± 11.40 μm, *p* < 0.001, Figure 1b, c). Masson’s trichrome staining revealed that the collagen content was significantly lower in the expanded skin’s dermis than in control skin’s dermis (34.06 ± 1.19% *vs* 46.52 ± 3.94%, *p* < 0.01, Figure 1b, d).

To determine the characteristics of S100A9 in expanded rat skin, we used RNA-seq analysis to detect its expression. The transcriptomic data revealed that S100A9 was overexpressed in expanded rat skin (Figure 1e). The qPCR results validated the elevated mRNA expression levels of S100A9 (*p* < 0.001, Figure 1f). Following the mRNA results, Western blot analysis confirmed increased protein expression of S100A9 in expanded rat skin (Figure 1g, h). A previous study showed that S100A9 functions by binding to the specific receptors—TLR-4 and/or RAGE [10]. Western blot results showed a higher protein expression of TLR-4 in the expanded rat skin (Figure 1g, i).

Exploring their protein expression and cellular locations will aid future research. Using immunofluorescence staining, we investigated the cellular localization of S100A9 and discovered that it was chiefly distributed in the cytoplasm of keratinocytes (Figure 1j). In addition, the average intensity of S100A9 (*p* < 0.01, Figure 1k) in the expanded epidermis of rats was higher than that in the control epidermis. Besides, immunofluorescence staining results revealed that TLR-4 was primarily located on the membrane of fibroblasts in the expanded dermis of rats; the average intensity of TLR-4 was significantly higher at the fibroblast surface in expanded rat skin than that in control rat skin (*p* < 0.01, Figure 1l, m).

**Figure 2:**
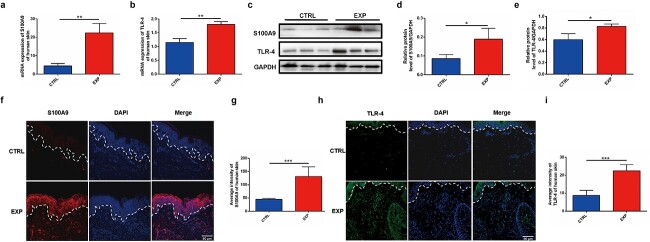
Increased expression of S100A9 and its receptor TLR-4 was observed in expanded human skin. (**a**) mRNA expression of S100A9 in expanded and control human skin (n = 3). (**b**) mRNA expression of TLR-4 in expanded and control human skin (n = 3). (**c**) Western blot results of S100A9 and TLR-4 expression in expanded and control human skin. (**d**) Western blot results of S100A9 expression in expanded and control human skin (n = 3). (**e**) The protein level of TLR-4 was increased in the expanded rat skin shown by Western blot (n = 3). (**f**) Immunofluorescence labelling revealed increased S100A9 expression in expanded human skin. (**g**) Quantification of the average intensity of S100A9 between the expanded and control human skin [n = 4 (normal skin), n = 11 (expanded skin)]. (**h**) Immunofluorescence staining analysis of TLR-4 expression in expanded human skin. (**i**) Quantification of the average intensity of TLR-4 between the expanded and control human skin (n = 3). ^*^p < 0.05, ^*^^*^p < 0.01, ^*^^*^^*^p < 0.001. *S100A9* S100 calcium-binding protein A9, *TLR-4* toll-like receptor 4, *CTRL* control, *EXP* expanded, *GAPDH* Glyceraldehyde-3-phosphate dehydrogenase

Furthermore, the expression and cellular location of S100A9 and TLR-4 were explored in expanded human skin. The qPCR results showed upregulated mRNA levels of S100A9 and TLR-4 (*p* < 0.01, Figure 2a, b). Western blot analysis further confirmed the increased expression of both proteins in human expanded skin (*p* < 0.05, Figure 2c, d and e). Immunofluorescence staining showed that S100A9 was found primarily in the epidermis of human expanded skin, with little or no S100A9 visible in the dermis (Figure 2f), and the average intensity of S100A9 was found to be significantly higher in the human-expanded epidermis compared to control skin (Figure 2g). These findings showed that S100A9 was primarily located in the keratinocytes of both rat and human-expanded epidermis and that mechanical stretch exerted by tissue expansion induced their expression. TLR-4 was mainly located on the cellular membrane of human fibroblasts and showed a higher average intensity in expanded human skin (*p* < 0.001, Figure 2h, i).

However, no differences were observed in the RAGE receptor between the expanded and control skin. Its expression was extremely low in both expanded and control human and rat skin (Figure S1, see online supplementary material), indicating that S100A9 functioned in the expanded skin by binding to TLR-4 of dermal fibroblasts. These results indicated that increased expression of S100A9 and TLR-4 was accompanied by decreased ECM in the expanded dermis.

### S100A9 was derived from stretched keratinocytes and facilitated human skin fibroblast proliferation and migration

**Figure 3:**
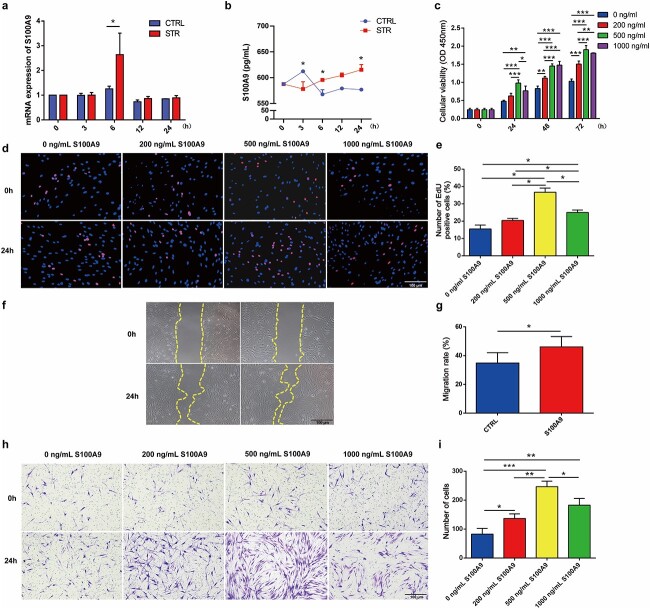
S100A9 was derived from stretched keratinocytes and facilitated human skin fibroblast proliferation and migration. (**a**) S100A9 mRNA expression in HaCaT cells was elevated after stretching. (**b**) Protein concentrations of S100A9 in the supernatants of stretched HaCaT cells at different intervals after stretching. (**c**) CCK-8 results of human skin fibroblasts treated with different concentrations of S100A9 at diverse time points. (**d**) Results of the 5-ethynyl-2′-deoxyuridine (EdU) proliferation assay performed on human skin fibroblasts treated with different concentrations of S100A9 at 24 h. (**e**) Proportion of EdU-positive cells. (**f**) Migration results of human skin fibroblasts treated with 500 ng/ml S100A9 at 24 h. (**g**) Quantification of fibroblast migration rate after 500 ng/ml S100A9 treatment. (**h**) Transwell assay of human skin fibroblasts treated with different concentrations of S100A9 at 24 h. (**i**) Number of migrating cells. ^*^p < 0.05, ^*^^*^p < 0.01, ^*^^*^^*^p < 0.001.* S100A9* S100 calcium-binding protein A9

An *in vivo* study showed that the mechanical stretch of tissue expansion upregulated the expression of S100A9. Accordingly, we stimulated the stretching force of tissue expansion to investigate its effects on S100A9 expression. There was a significant increase in the mRNA expression of S100A9 (*p* < 0.05, Figure 3a) after 6 h of stretching. The protein levels of S100A9 in the supernatants of stretched HaCaT cells were measured using an ELISA. Following stretching, compared with the supernatant of control cells, the supernatant of HaCaT cells showed higher levels of S100A9 at 6 and 24 h (*p* < 0.05, Figure 3b), indicating that S100A9 played important paracrine roles in the expanded dermis where the receptor TLR-4 was highly expressed on fibroblast membranes.

S100A9 was primarily found in mechanically stretched epidermal keratinocytes, whereas its receptor TLR-4 was primarily observed in expanded dermal fibroblasts. An *in vitro* study revealed that mechanical stretch increased S100A9 expression in keratinocytes and protein secretion into the cell culture supernatants. These findings suggested that S100A9 plays a role in regulating fibroblasts, thereby contributing to increased skin regeneration. Therefore, we investigated the effects of S100A9 on human skin fibroblasts. The CCK-8 results showed that compared with the control group, more robust fibroblast proliferation was observed at 24, 48 and 72 h after treatment with different concentrations of S100A9; CCK-8 and EdU proliferation assay results both showed that 500 ng/ml of S100A9 treatment exhibited the best effects to promote cell proliferation (Figure 3c–e). Similarly, S100A9 treatment (500 ng/ml) effectively promoted human skin fibroblast migration (Figure 3f–i).

### S100A9 promoted human skin fibroblast proliferation and migration and COL I and TGF-β expression via the TLR-4/ERK1/2 pathway

**Figure 4:**
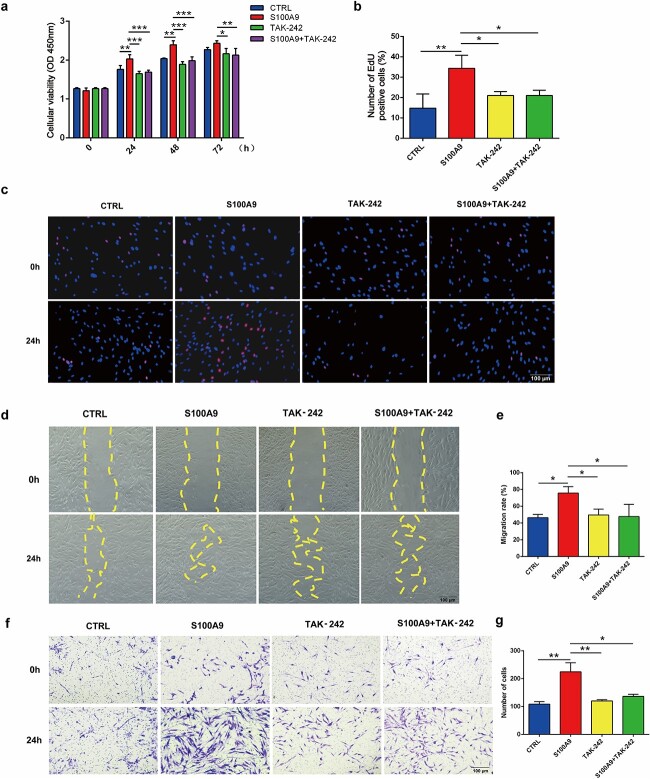
S100A9 promoted human skin fibroblast proliferation and migration in a TLR-4 dependent manner. (**a**) CCK8 results of human skin fibroblasts treated with S100A9 and 1 μM TAK-242 at diverse time points. (**b**) Proportions of EdU-positive cells treated with 1 μM TAK-242 at 24 h. (**c**) Representative images of EdU positive fibroblasts treated with 1 μM TAK-242. (**d**) Representative migration pictures of human fibroblasts treated with 1 μM TAK-242. (**e**) Quantification of fibroblast migration rate after treatment with 1 μM TAK-242 for 24 h. (**f**) Transwell assay of human skin fibroblasts treated with 1 μM TAK-242. (**g**) Number of migrating cells. ^*^p < 0.05, ^*^^*^p < 0.01, ^*^^*^^*^p < 0.001.* S100A9* S100 calcium-binding protein A9, *TLR-4* toll-like receptor 4

As demonstrated in quantitative analysis of the average intensity in the expanded and control skin, the protein expression of S100A9 and its receptor TLR-4 were elevated. To assert whether S100A9 could promote the expression of COL I and TGF-β by binding to TLR-4, we used TAK-242, an inhibitor of TLR-4. The cytotoxicity effects of TAK-242 of diverse concentrations were detected using CCK8 assay; the results showed that 1 μM TAK-242 was safe for cells and could be used for further study (Figure S2, see online supplementary material). CCK-8 (Figure 4a), EdU proliferation (Figure 4b, c), wound healing (Figure 4d, e) and transwell assays (Figure 4f, g) further revealed that S100A9 treatment accelerated fibroblast proliferation and migration, whereas TAK-242 inhibited these effects of S100A9 (Figure 4).

**Figure 5:**
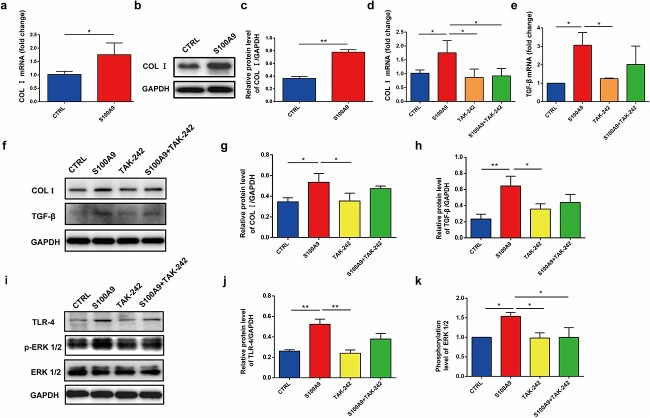
S100A9 promoted human skin fibroblast collagen I (COL I) and transforming growth factor beta (TGF-β) expression through the TLR-4/extracellular signal-regulated kinase (ERK) 1/2 pathway. (**a**) COL I mRNA expression in control fibroblasts and S100A9-treated fibroblasts. (**b**) COL I protein levels in skin fibroblasts from control and S100A9-treated fibroblasts were detected using Western blot. (**c**) Quantification of protein levels of COL I in control and S100A9-treated fibroblasts. (**d**) COL I mRNA expression in control, S100A9-treated, TAK-242-treated and S100A9 + TAK-242-treated fibroblasts. (**e**) TGF-β mRNA expressions in control, S100A9-treated, TAK-242–treated and S100A9 + TAK-242–treated fibroblasts. (**f**) Western blot examination of skin fibroblasts from control, S100A9-treated, TAK-242–treated and S100A9 + TAK-242–treated fibroblasts revealed the protein levels of COL I and TGF-β. (**g**) Quantification of protein levels of COL I in control, S100A9-treated, TAK-242-treated and S100A9 + TAK-242-treated fibroblasts. (**h**) Quantification of protein levels of TGF-β in control, S100A9-treated, TAK-242-treated and S100A9 + TAK-242-treated fibroblasts. (**i**) Protein levels of TLR-4, ERK1/2 and p-ERK1/2 of skin fibroblasts from control, S100A9-treated, TAK-242-treated and S100A9 + TAK-242-treated fibroblasts detected by Western blot. (**j**) Quantification of protein levels of TLR-4 in control, S100A9-treated, TAK-242-treated and S100A9 + TAK-242-treated fibroblasts. (**k**) Quantification of the phosphorylation levels of ERK1/2 in control, S100A9-treated, TAK-242-treated and S100A9 + TAK-242-treated fibroblasts. ^*^p < 0.05, ^*^^*^p < 0.01. *S100A9* S100 calcium-binding protein A9

In addition to the proliferation and migration assays, additional experiments were conducted to characterize fibrosis properties. S100A9-treated skin fibroblasts expressed higher mRNA (*p* < 0.05, Figure 5a) and protein (*p* < 0.01, Figure 5b, c) levels of COL I. The qPCR and Western blot findings showed that COL I and TGF-β levels were significantly induced by S100A9 treatment. TLR-4 protein levels were altered in response to this treatment. TLR-4 inhibition by TAK-242 reversed the increased expression of downstream genes such as COL I and TGF-β, indicating that S100A9 upregulated the expressions of COL I and TGF-β by binding to the receptor TLR-4 (Figure 5d–h). Furthermore, TLR-4 increased the phosphorylation of ERK1/2 (Figure 5i, j and k). These findings suggest that S100A9 promotes the expression of COL I and TGF-β in human skin fibroblasts via the TLR-4/ERK1/2 pathway.

### S100A9 secreted from mechanically stretched HaCaT cells stimulated skin fibroblast COL I, TGF-β, TLR-4 and p-ERK1/2 expression

**Figure 6:**
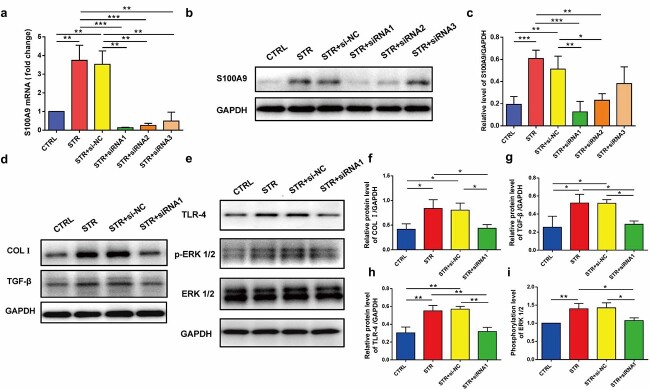
S100A9 secreted from mechanically stretched HaCaT cells stimulated collagen I (COL I), transforming growth factor beta (TGF-β), toll-like receptor 4 (TLR-4) and p-extracellular signal-regulated kinase (ERK)1/2 expression in fibroblasts. (**a**) S100A9 mRNA expression in control cells (CTRL), stretched HaCaT cells (STR), negative control siRNA-transfected stretched HaCaT cells (STR + si-NC) and S100A9 siRNA-transfected stretched HaCaT cells (STR + siRNA). (**b**) Western blot study of the S100A9 protein levels in CTRL, STR, STR + si-NC and STR + siRNA cells. (**c**) Quantification of S100A9 protein levels of CTRL, STR, STR + si-NC and STR + siRNA cells using Western blot analysis. (**d**) Protein levels of COL I and TGF-β in fibroblasts treated with the supernatants of CTRL, STR, STR + si-NC and STR + siRNA cells detected using Western blot analysis. (**e**) Protein levels of TLR-4, ERK1/2 and p-ERK in fibroblasts treated with supernatants of CTRL, STR, STR + si-NC and STR + siRNA cells detected by Western blot analysis. (**f**) Quantification of COL I protein levels in fibroblasts treated with supernatants of CTRL, STR, STR + si-NC and STR + siRNA cells. (**g**) Quantification of TGF-β protein levels in fibroblasts treated with supernatants of CTRL, STR, STR + si-NC and STR + siRNA cells. (**h**) Quantification of the TLR-4 protein levels in fibroblasts treated with the supernatants of CTRL, STR, STR + si-NC and STR + siRNA cells. (**i**) Quantification of phosphorylated ERK1/2 in fibroblasts treated with supernatants of CTRL, STR, STR + si-NC and STR + siRNA cells. ^*^p < 0.05, ^*^^*^p < 0.01, ^*^^*^^*^p < 0.001. *S100A9* S100 calcium-binding protein A9, *GAPDH* Glyceraldehyde-3-phosphate dehydrogenase

Based on our hypothesis that S100A9 secreted from keratinocytes played essential roles via a paracrine mode in the dermis where the S100A9 receptor was expressed on fibroblast membranes, we co-cultured HaCaT cells with human skin fibroblasts to confirm the profibrotic effects of S100A9 on fibroblasts. First, we transfected HaCaT cells with S100A9 siRNAs that inhibited S100A9 expression; we selected siRNA1 for the highest levels of gene silencing for further studies (Figure 6a, b and c). The fibroblasts cultured with the supernatants of stretched HaCaT cells and negative control siRNA-transfected stretched HaCaT cells showed higher levels of COL I and TGF-β; by contrast, S100A9 knockdown with S100A9 siRNA1 reversed the stretching-induced COL I and TGF-β expression in these cells. Furthermore, there was no significant difference in the expressions of COL I and TGF-β in fibroblasts cultured with the supernatants of control cells and S100A9 siRNA1-transfected HaCaT cells (Figure 6d, f and g). Moreover, TLR-4 and p-ERK1/2 protein levels were altered (Figure 6e, h and i).

### Recombined S100A9 protein benefited expanded rat skin regeneration and rescued dermal thinning *in vivo*

**Figure 7:**
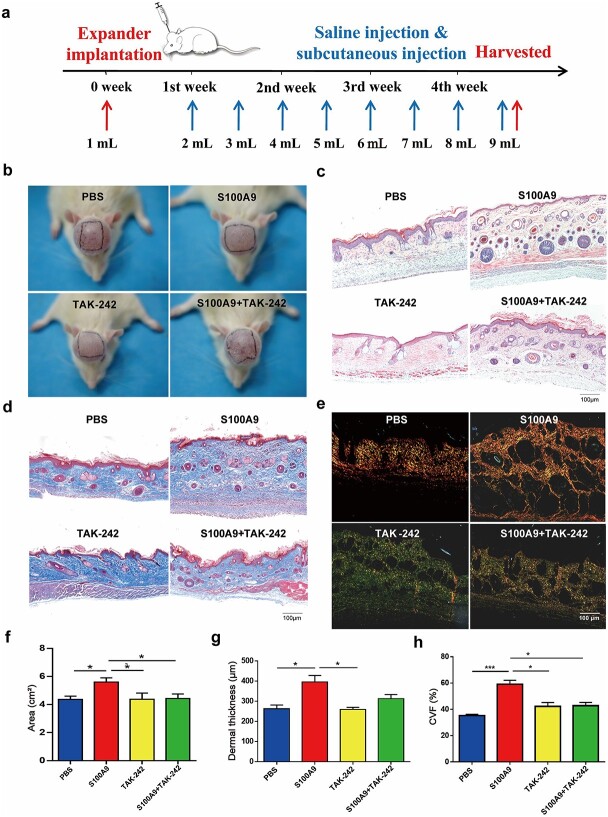
Recombined S100A9 protein benefited rat skin regeneration and rescued dermal thinning *in vivo*. (**a**) Administration of various treatments in rats during tissue expansion. (**b**) Representative expansion areas of PBS-treated, S100A9-treated, TAK-242–treated and S100A9 + TAK-242–treated rat skin. (**c**). Haematoxylin and eosin-stained images of PBS-treated, S100A9-treated, TAK-242–treated and S100A9 + TAK-242-treated skins. (**d**) Masson’s trichrome-stained images of PBS-treated, S100A9-treated, TAK-242–treated, and S100A9 + TAK-242-treated skins. (**e**) Sirius red-stained images of PBS-treated, S100A9-treated, TAK-242-treated and S100A9 + TAK-242-treated skins; the red or yellow areas represent COL I, whereas the green area represents COL III. (**f**) Quantification of expansion areas following the administration of various treatments to the expanded skin (n = 4). (**g**) Quantification of dermal thickness following different treatments (n = 4). (**h**) Quantification of collagen content described as collagen volume fraction (CVF) following the administration of various treatments to expanded rat skin (n = 4). ^*^p < 0.05, ^*^^*^^*^p < 0.001. *S100A9* S100 calcium-binding protein A9, *PBS* phosphate-buffered solution

The rat scalp expansion model was set up as previously described; each treatment was followed by expander enlargement (Figure 7a). Following the injection of the expanders with saline for 4 weeks, the tattooed areas were measured(Figure 7b), and H&E staining (Figure 7c) together with Masson’s trichrome staining were represented (Figure 7d). Sirius red staining revealed that S100A9 increased dermal COL I levels, which was denoted as red or yellow colouration (Figure 7e).

Specifically, the S100A9 group exhibited a larger tattooed flap area (5.59 ± 0.31 cm^2^) compared with the PBS-treated group (4.36 ± 0.24 cm^2^), TAK-242–treated skin (4.38 ± 0.44 cm^2^) and S100A9 + TAK-242–treated skin (4.45 ± 0.45 cm^2^; Figure 7f). The effects of S100A9 on the expanded dermis were studied using expanded dermal thickness and collagen content. Remarkably, H&E image examination revealed that S100A9-treated skin in the expansion group (441.10 ± 44.97 μm) possessed thicker dermal thickness compared with PBS-treated skin (262.50 ± 46.65 μm), TAK-242-treated skin (259.50 ± 20.74 μm) and S100A9 + TAK-242-treated skin (313.00 ± 50.56 μm; Figure 7g). Masson’s trichrome staining revealed that collagen content was significantly higher in the dermis of S100A9-treated expanded skin (51.40 ± 17.32%) than in that of PBS-treated skin (35.23 ± 2.46%), TAK-242-treated skin (42.35 ± 7.17%) and S100A9 + TAK-242-treated skin (42.74 ± 4.27%; Figure 7d, h).

### Recombined S100A9 protein enhanced ECM deposition in expanded rat skin

**Figure 8:**
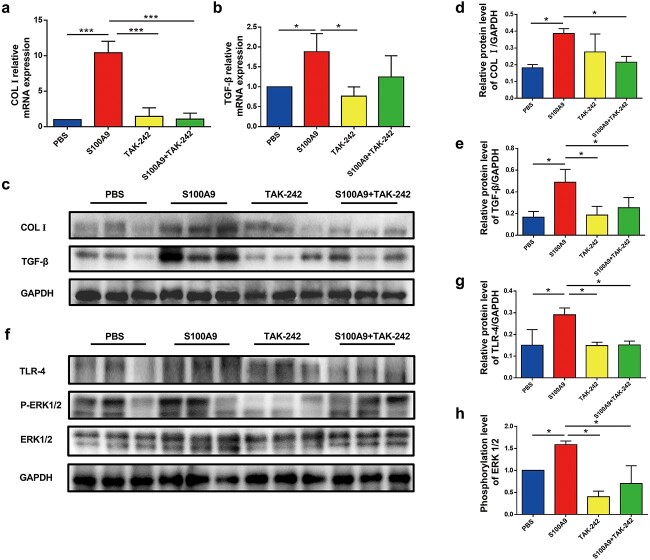
Recombined S100A9 protein enhanced extracellular matrix (ECM) deposition in expanded rat skin. (**a**) Collagen I (COL I) mRNA expressions in PBS-treated, S100A9-treated, TAK-242–treated and S100A9 + TAK-242-treated skins (n = 3). (**b**) Transforming growth factor beta-β (TGF-β) mRNA expression in PBS-treated, S100A9-treated, TAK-242-treated and S100A9 + TAK-242-treated skins (n = 3). (**c**) The protein levels of COL I and TGF-β in PBS-treated, S100A9-treated, TAK-242-treated and S100A9 + TAK-242-treated skins detected using Western blot. (**d**) Quantification of the COL I protein levels in PBS-treated, S100A9-treated, TAK-242-treated and S100A9 + TAK-242-treated skins (n = 3). (**e**) Quantification of the TGF-β protein levels in PBS-treated, S100A9-treated, TAK-242-treated and S100A9 + TAK-242-treated skins (n = 3). (**f**) Protein levels of TLR-4, ERK1/2 and p-ERK1/2 in PBS-treated, S100A9-treated, TAK-242-treated and S100A9 + TAK-242-treated skin detected using Western blot analysis. (**g**) Quantification of protein levels of TLR-4 in PBS-treated, S100A9-treated, TAK-242-treated and S100A9 + TAK-242-treated skins (n = 3). (**h**) Quantification of the phosphorylation levels of ERK1/2 in PBS-treated, S100A9-treated, TAK-242-treated and S100A9 + TAK-242-treated skins (n = 3). ^*^p< 0.05, ^*^^*^^*^p < 0.001. *S100A9* S100 calcium-binding protein A9, *PBS* phosphate-buffered solution, *TLR-4* toll-like receptor 4, *GAPDH* Glyceraldehyde-3-phosphate dehydrogenase

To investigate the effects of recombined S100A9 protein on ECM deposition, we measured the mRNA and protein levels of COL I and TGF-β in S100A9-treated expanded rat skin. Compared with the S100A9-treated skin, the expression of these genes was reduced in all the remaining three groups (Figure 8a–e). Considering the complex microenvironment *in vivo*, S100A9 treatment may result in slightly increased expression of protein such as TGF-β. However, there were no significant differences observed between the TAK-242-treated and S100A9 + TAK-242-treated groups. This suggested that the TLR-4 inhibitor TAK-242 played a role in the profibrotic effects of S100A9. TLR-4 protein levels were altered to correspond to these proteins. S100A9 reportedly activates the mitogen-activated protein kinase (MAPK) and nuclear factor-κB (NF-κB) pathways [27–29]. The protein levels of P38, p-P38, c-Jun N-terminal kinase (JNK) and phospho-JNK were examined in the expanded skin, but no significant differences were observed (data not shown). These results demonstrated that S100A9 could cause ERK1/2 phosphorylation in tissues, indicating that S100A9 binding to TLR-4 could activate the ERK1/2 signalling pathway (Figure 8f–h).

## Discussion

Tissue expansion provides patients with additional skin for defect repair. However, issues such as low expansion efficiency and skin rupture challenge the application of this technique [30]. To address these issues, we analysed the expression and functions of S100A9 and observed that S100A9 expression was elevated in expanded human and rat epidermis. Exogenous S100A9 protein could increase expanded skin areas and dermal thicknesses as well as increase ECM deposition *in vivo* by binding to the TLR-4 receptor and activating the ERK1/2 signalling pathway. *In vitro*, S100A9 was increased in stretched HaCaT cells. S100A9 accelerated human skin fibroblast proliferation, migration and collagen synthesis. Furthermore, the co-culture of fibroblasts with the supernatants of stretched HaCaT cells induced a higher expression of collagen in skin fibroblasts, indicating that S100A9 synthesised and secreted from expanded epidermal keratinocytes may function in promoting expanded dermal regeneration via a paracrine mode by binding to fibroblast TLR-4.

S100A9 is a low-molecular-weight protein that participates in multiple cellular processes [31, 32]. Typically, it is abundantly expressed in myeloid cells but not in normal skin [33]. However, as demonstrated in the present study, S100A9 was primarily located in the expanded epidermis, consistent with previous research using single-cell resolution analysis [20]. Considering that the skin experiences repeated stress and relaxation and injury and repair during tissue expansion [34], it can be believed that S100A9 is activated by mechanical stretch and participates in tissue repair. S100A9 overexpression was also reported to be associated with other skin diseases, such as psoriasis [35]. In another study, decreased hydration was found to increase S100A9 expression in epidermal keratinocytes [17]. These findings indicate that S100A9 is a critical skin and tissue repair regulator.

Several studies have shown that cytokines play a role in cellular processes contributing to skin regeneration during tissue expansion. S100A9 is essential for ECM deposition [36, 37]. In pulmonary diseases, active roles of S100A9 include fibroblast proliferation and collagen production upregulation [16]. S100A9-knockout mice possessed fewer myofibroblasts and exhibited less kidney collagen deposition [13]. Consistent with these observations, the findings of the present study revealed the fibrotic effects during skin expansion. TGF-β was increased in addition to collagen synthesis, which directly activated fibroblasts to differentiate into myofibroblasts [13]. *In vitro* studies have revealed that S100A9 promoted fibroblast proliferation and migration. All these factors contribute to dermal thickening because the dermis primarily comprises ECM and fibroblasts. These findings suggested that the *in vivo* application of S100A9 promoted dermal thickening and skin regeneration during tissue expansion.

However, dermal thinning was accompanied by an increase in endogenous S100A9 expression during growth; this was attributed to two factors. On the one hand, tissue expansion resulted in dermal thinning and repeated skin injuries. Endogenous S100A9 protein was gradually secreted under this microenvironment and acted as a ‘fireman’ to rescue dermal thinning. However, dermal thinning could not be reversed at the time. On the other hand, endogenous S100A9 was insufficient to completely reverse dermal thinning. To test our hypothesis, we injected the expanded skin with recombined S100A9 protein. The expanded dermis thickened as expected, possibly owing to the increased S100A9 protein level in the expanded skin. These findings suggested that measures to facilitate endogenous S100A9 secretion in expanded skin should be implemented at the earliest opportunity to promote skin regeneration. Moreover, using exogenous S100A9 protein during skin expansion would be beneficial.

Secreted S100A9 binds to the receptors TLR-4 [38, 39] and RAGE [40, 41]. TLR-4 is a member of the Toll-like receptor family and recognises and binds to various molecular patterns to initiate intracellular signalling pathways [10]. RAGE belongs to the immunoglobulin superfamily [42]. Remarkably, S100A9 binds to both RAGE and TLR-4, which possess several common ligands and downstream signalling pathways. However, TLR-4 was primarily expressed and located on fibroblast membranes in the expanded skin, whereas RAGE expression was extremely low. Therefore, we focused on TLR-4 in this study. In hypertrophic scar and keloid tissues, S100A9 secreted by epidermal keratinocytes activated dermal fibroblasts in the skin [18, 43, 44]. Similarly, secreted S100A9 from the expanded epidermis could bind to TLR-4 and play a role in ECM deposition in a paracrine mode. TLR-4 deficiency reportedly reduced tissue fibrosis [15]. To confirm the role of TLR-4 in expanded skin, we injected its specific inhibitor TAK-242 into expanded rat skin. We observed that S100A9 no longer exerted its profibrotic effects following TAK-242 injection, consistent with the *in vitro* findings. S100A9 promoted ERK1/2 phosphorylation in human lung fibroblasts [27]. Furthermore, S100A9 exacerbated skin fibrosis and increased the expression of ERK1/2 MAPK signalling pathways in scleroderma [45]. Previous research has demonstrated that S100A9 could activate the p38, JNK or NF-κB pathways. However, we did not observe similar results because only TLR-4 was activated; this could be attributed to the unique microenvironment of mechanical stretch in the expanded skin. Furthermore, S100A9 could interact with TLR-4 to promote cell growth [46], thereby influencing the activation of downstream transcription factors such as Jun [47] and Fos [48]. Because S100A9 has been shown to promote tissue ECM deposition via the TLR-4/ERK1/2 pathway during skin expansion, the downstream regulator may be investigated.

## Conclusions

The findings of the present study demonstrated that S100A9 could be activated by mechanical stretch during tissue expansion; its activation alleviated dermal thinning, increased skin area, and improved tissue ECM production by binding to the TLR-4 receptor and activating ERK1/2. Therefore, S100A9 could be a promising intervention target or medication for reversing dermal thinning and promoting skin regeneration during tissue expansion.

## Supplementary Material

Figure_S1_tkad030

Figure_S2_tkad030

Table_S1_tkad030

Table_S2_tkad030

## Data Availability

This published article includes all data generated or analysed during this study. The RNA-Seq datasets used in this study are available in online repositories. The repository/repository names and accession number(s) are listed below: NCBI SRA BioProject, accession no: PRJNA753961.

## Abbreviations

ANOVA: Analysis of variance; BCA: Bicinchoninic acid assay; CCK-8: Cell counting kit-8; COL I: Collagen I; CVF: Collagen volume fraction; ECM: Extracellular matrix; Edu: 5-Ethynyl-2′-deoxyuridine; ELISA: Enzyme-linked immunosorbent assay; GAPDH: Glyceraldehyde-3-phosphate dehydrogenase; H&E: Haematoxylin and eosin; JNK: c-Jun N-terminal kinase; MAPK: Mitogen-activated protein kinase; NF-κB: Nuclear factor-κB; PBS: Phosphate-buffered solution; p-ERK1/2: Phospho-extracellular signal-related kinase 1/2; qPCR: Quantitative polymerase chain reaction; RAGE: Receptor for advanced glycation end products; RNA-seq: Single-cell RNA sequencing; siRNA: Small interfering RNA; S100A9: S100 calcium-binding protein A9; TGF-β: Transforming growth factor beta; TLR-4: Toll-like receptor 4.
