# Supplementary figures and images for: S100 calcium-binding protein A9 promotes skin regeneration through toll-like receptor 4 during tissue expansion
Source: Burns Trauma. 2023 Oct 31;11:tkad030. doi: 10.1093/burnst/tkad030 (PMC10627002; doi:10.1093/burnst/tkad030)

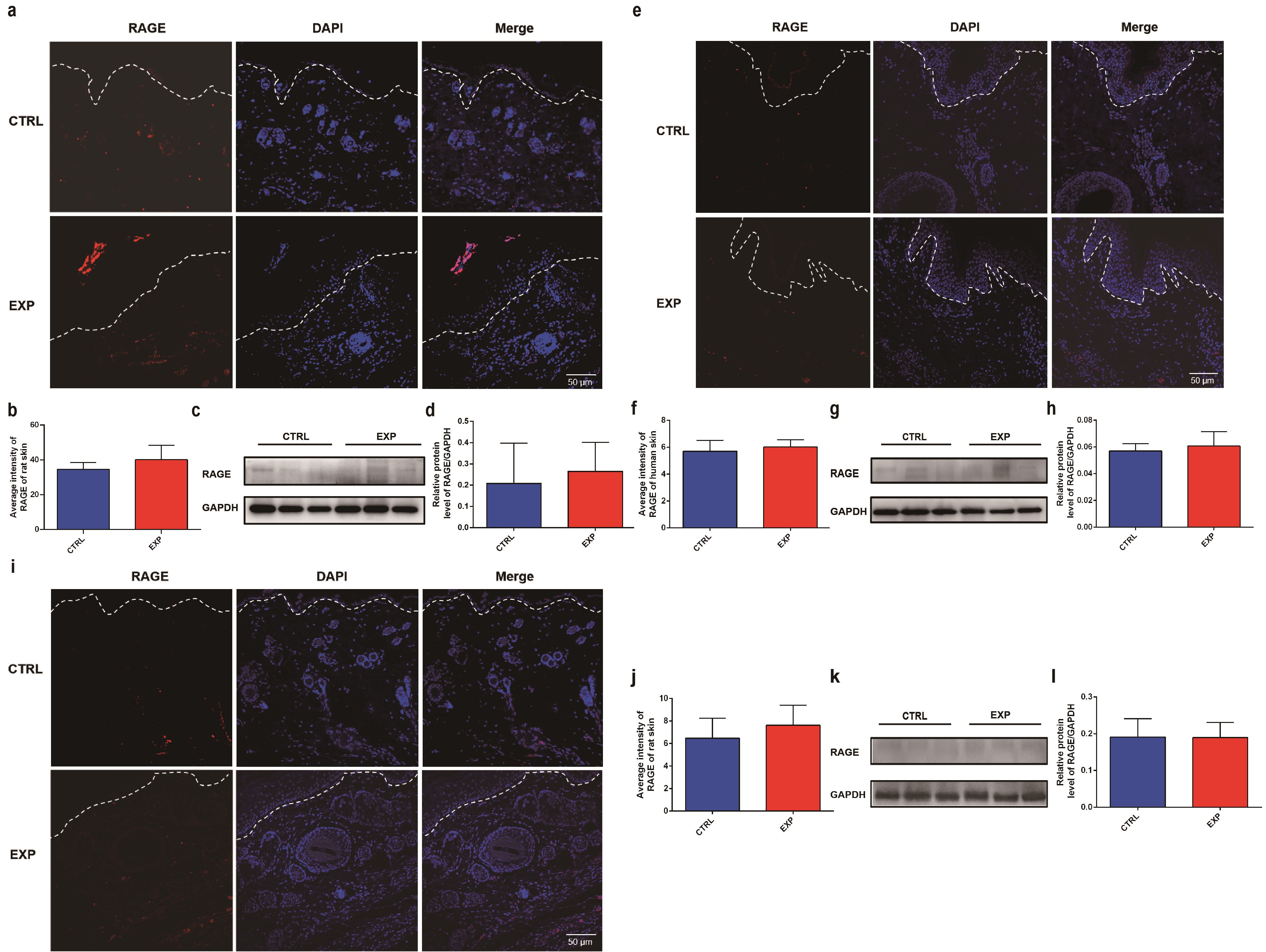

Supplement: Figure_S1_tkad030 [file figure_s1_tkad030.jpeg]

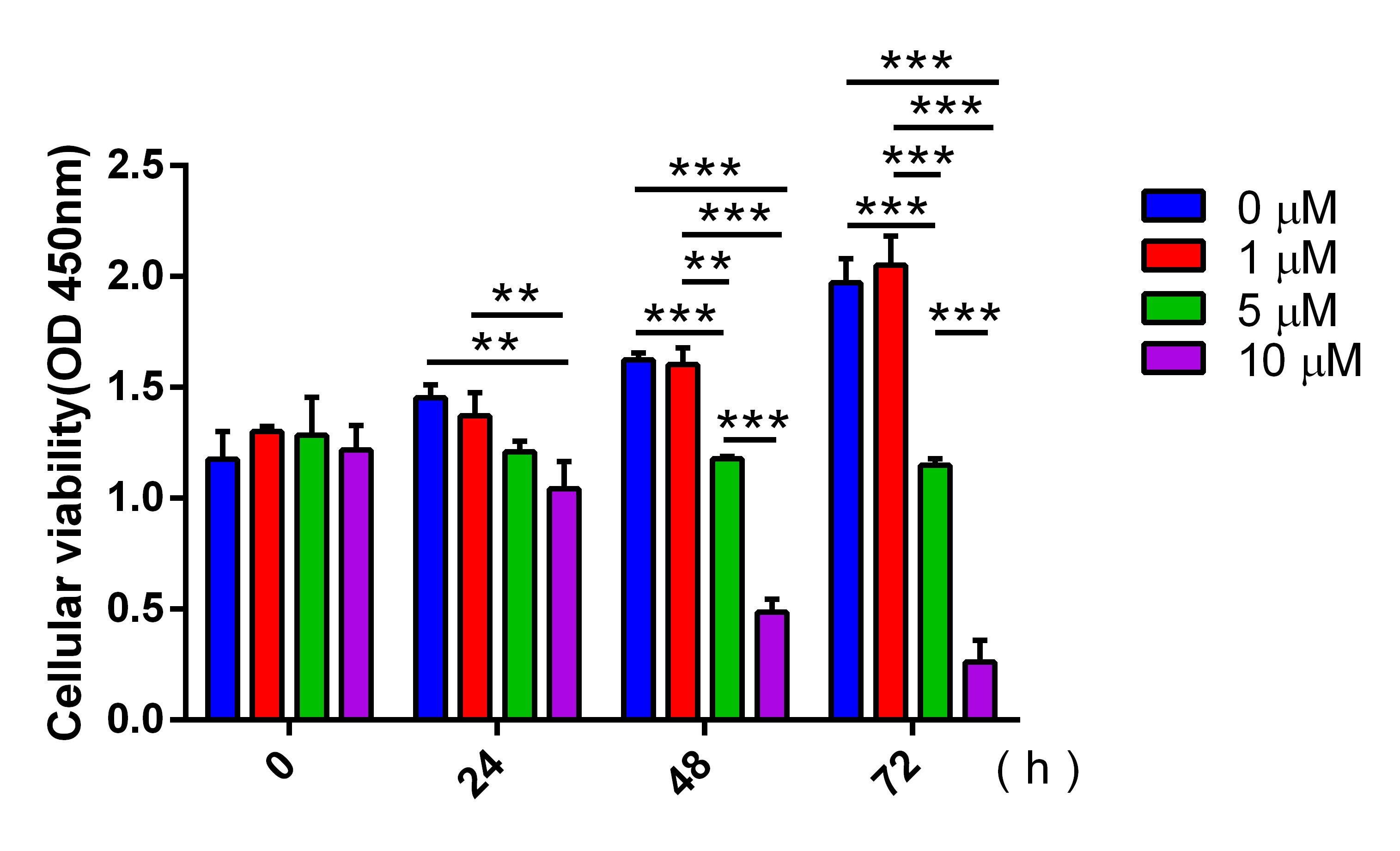

Supplement: Figure_S2_tkad030 [file figure_s2_tkad030.jpeg]
