# Supplementary material for: S100 calcium-binding protein A9 promotes skin regeneration through toll-like receptor 4 during tissue expansion
Source: Burns Trauma. 2023 Oct 31;11:tkad030. doi: 10.1093/burnst/tkad030 (PMC10627002; doi:10.1093/burnst/tkad030)
Supplement: Table_S1_tkad030 [file table_s1_tkad030.docx]

**Table S1. Clinical information for human skin samples**

| **Patient** | **Placed area** | **Time (d)** | **Gender** | **Age** | **Volume of tissue expander (mL)** | **Explanation** |
| --- | --- | --- | --- | --- | --- | --- |
| 1 | Right ear | 84 | Male | 15 | 130 | Expanded skin and normal skin |
| 2 | Left ear | 91 | Female | 17 | 126 | Expanded skin and normal skin |
| 3 | Right ear | 73 | Male | 12 | 130 | Expanded skin and normal skin |
| 4 | Right ear | 101 | Male | 12 | 113 | Expanded skin and normal skin |
| 5 | Left ear | 88 | Male | 22 | 120 | Expanded skin |
| 6 | Right ear | 83 | Male | 10 | 121 | Expanded skin |
| 7 | Right ear | 89 | Male | 12 | 126 | Expanded skin |
| 8 | Right ear | 82 | Male | 11 | 115 | Expanded skin |
| 9 | Right ear | 92 | Male | 13 | 121 | Expanded skin |
| 10 | Right ear | 87 | Female | 12 | 121 | Expanded skin |
| 11 | Left ear | 92 | Male | 25 | 120 | Expanded skin |
