# Supplementary material for: S100 calcium-binding protein A9 promotes skin regeneration through toll-like receptor 4 during tissue expansion
Source: Burns Trauma. 2023 Oct 31;11:tkad030. doi: 10.1093/burnst/tkad030 (PMC10627002; doi:10.1093/burnst/tkad030)
Supplement: Table_S2_tkad030 [file table_s2_tkad030.docx]

**Table S2. Primer pairs used for Real-time PCR**

| **Target gene** | **Forward (5’-3’)** | **Reverse (5’-3’)** |
| --- | --- | --- |
| *rS100A9* | CAGCATAAGCACCATCATCAA | TTCCCATCAGCATCATACACTC |
| *rCOL Ⅰ* | TGACTGGAAGAGCGGAGAGT | GATAGCGACATCGGCAGGAT |
| *rTGF-β* | GCTGAACCAAGGAGACGGAAT | AGGTGTTGAGCCCTTTCCAG |
| *rGAPDH* | AAGATCGGAATTAACGGATTTGGC | GCCCTTGAAACGACCGTGAGT |
| *hS100A9* | GGTCATAGAACACATCATGGAGG | GGCCTGGCTTATGGTGGTG |
| *hCOL Ⅰ* | TCTGCGACAACGGCAAGGTG | GACGCCGGTGGTTTCTTGGT |
| *hTGF-β* | CGA GCCTGAGGCCGACTAC | AGATTTCGTTGTGGGTTTCCA |
| *hGAPDH* | TGTTGCCATCAATGACCCCTT | CTCCACGACGTACTCAGCG |
